# Perillaldehyde Controls Postharvest Black Rot Caused by *Ceratocystis fimbriata* in Sweet Potatoes

**DOI:** 10.3389/fmicb.2018.01102

**Published:** 2018-05-25

**Authors:** Man Zhang, Man Liu, Shenyuan Pan, Chao Pan, Yongxin Li, Jun Tian

**Affiliations:** ^1^College of Life Science, Jiangsu Normal University, Xuzhou, China; ^2^Beijing Advanced Innovation Center for Food Nutrition and Human Health, Beijing Technology and Business University, Beijing, China

**Keywords:** *Perilla*, postharvest disease, antifungal, enzyme activity, fruit quality

## Abstract

Black rot caused by *Ceratocystis fimbriata* is the most damaging postharvest disease among sweet potatoes. Black rot can be controlled by synthetic fungicides, but these synthetic fungicides also have several negative effects. Perillaldehyde (PAE), a major component of the herb perilla, is an effective and eco-friendly method of controlling this disease. The antifungal activity of PAE on the mycelial growth in *C. fimbriata* was evaluated *in vitro*. Sweet potatoes at the postharvest stage were surfaced-disinfected with 75% ethanol. Artificially created wounds were inoculated with a *C. fimbriata* cell suspension, and then, the PAE was spontaneously volatilized inside the residual airspace of the containers at 28°C. Samples were collected at 0, 3, 6, 9, 12, 15, 18, and 21 days from each group, and the tissues around the wounds of the sweet potatoes were collected using a sterilized knife and then homogenized to determine their defense-related enzyme activity and quality parameters. *In vitro* assays showed that the mycelial growth of *C. fimbriata* was inhibited by PAE in a dose-dependent manner. An *in vivo* test demonstrated that 25, 50, and 100 μl/l PAE doses, when applied to sweet potatoes inoculated with *C. fimbriata*, could remarkable lower lesion diameter as compared to the control. Even though the storage time was prolonged, PAE vapor treatment still drastically inhibited sweet potato decay during storage at 28°C. These PAE vapor treatments also enhanced the activities of superoxide dismutase (SOD), catalase (CAT), ascorbate peroxidase (APX), peroxidase (POD), polyphenol oxidase (PPO), and phenylalanine ammonia-lyase (PAL). These treatments remarkably decreased weight loss rates and had minor effects on other fruit quality parameters, such as anthocyanin content and vitamin C content. In our study, the results suggested that the effects of PAE on postharvest sweet potatoes may be attributed to the maintenance of enzymatic activity and fruit quality. In sum, PAE may be a promising approach to controlling *C. fimbriata* in sweet potatoes.

## Introduction

*Ceratocystis fimbriata* is a pathogenic fungus that causes lethal wilt-type diseases in a broad range of economically important plants (Ferreira et al., 2017). *C. fimbriata* on sweet potatoes [*Ipomoea batatas* (L.) Lam.] was first reported in China, where there were substantial losses due to black rot on stored roots (Muramoto et al., 2012). A widely distributed strain of *C. fimbriata* has been reported to cause black rot in sweet potatoes and severe deterioration during postharvest storage (Baker et al., 2003; Engelbrecht and Harrington, 2005). Postharvest diseases of crops and fruits cause major losses, and these diseases are primarily controlled via the application of synthetic fungicides. However, in recent years, the resistance of *C. fimbriata* to conventional synthetic fungicides has drastically increased due to the fact that the widespread, long-term agricultural use of synthetic fungicides has caused some major postharvest pathogens to develop resistance against them (Vilaplana et al., 2017). Also, there is a current trend toward safer and more eco-friendly fungicides for the control of postharvest decay (Sharma et al., 2009). Hence, the development of more effective and healthy antifungals is of paramount importance.

Essential oils (EOs) have been used for 1000s of years in food preservation pharmaceuticals and alternative medicine and have attracted interest due to their relative safety, volatility, broad acceptance by consumers, and eco-friendliness (Prabuseenivasan et al., 2006; Tzortzakis and Economakis, 2007; Liu et al., 2016; Servili et al., 2017). Numerous studies have reported antifungal effects on the part of various EOs used to control deterioration in postharvest fruits and vegetables (Soylu et al., 2010; Fan et al., 2014; Elshafie et al., 2015; Guerra et al., 2015). Perillaldehyde (PAE), a major constituent of essential oil, is found most abundantly in the herb perilla (*Perilla frutescens*, Labiatae), which has been widely used as a medicinal agent (Hobbs et al., 2016). PAE is a safe flavoring additive in foods and a safe ingredient in perfume (Wang et al., 2008). PAE exhibits antioxidant, antidepressant, and other biological properties and also shows antimicrobial activity against *Candida albicans, Aspergillus flavus, A. niger*, and other microbes (McGeady et al., 2002; Tian et al., 2015a, 2016, 2017). In addition, it can also be developed into a natural preservative to control postharvest fungal decay in table grapes and cherry tomatoes (Tian et al., 2015a,b).

However, information on the effect of PAE on the postharvest activity of defense-related enzymes and fruit quality in crops is lacking. More importantly, the purpose of our study is to investigate the postharvest application of PAE as a novel strategy for the control of postharvest diseases in sweet potatoes. Therefore, in this study, we aimed to evaluate the antifungal activity of PAE against *C. fimbriata* through *in vitro* and *in vivo* experiments and to determine the influences of PAE on the defense-related activity of several enzymes, including superoxide dismutase (SOD), catalase (CAT), ascorbate peroxidase (APX), peroxidase (POD), polyphenol oxidase (PPO), and phenylalanine ammonialyase (PAL), in sweet potatoes, as well as on certain fruit quality parameters, such as weight loss, anthocyanin content, and vitamin C content.

## Materials and Methods

### Medicament, Pathogen, and Plant Materials

The PAE was prepared as a stock solution in 0.1% (v/v) Tween 80. The isolates of *C. fimbriata* (voucher specimen number CF1.01127) used in this work were obtained from spoiled sweet potatoes (*Ipomoea batatas* Lam. cv. Sushu 8) in a greenhouse at Xuzhou Academy of Agricultural Sciences and then identified via morphological and molecular biology techniques. They were preserved on potato dextrose agar (PDA) that contained an infusion of 200 g/l potatoes, 20 g/l glucose, and 20 g/l agar at 28°C. The spores from a 7-day-old culture were suspended in 0.1% (v/v) Tween 80 and adjusted to 10^6^ spores/ml using a hemacytometer. Sweet potatoes were harvested and removed from a commercial greenhouse around Jiangsu Normal University and transported to the laboratory within 2 h. Healthy sweet potatoes of uniform size and maturation were chosen for the experiments.

### *In Vitro* Assay

The inhibition of mycelial growth was analyzed using a modified version of the method of Soylu et al. (2006) and Shao et al. (2013). Glass Petri dishes (90 mm × 20 mm, with 80 ml air spaces after the addition of 20 ml of agar media) were filled with 20 ml of PDA, and a mycelial disk (6 mm in diameter) was placed in the center of each plate. Next, the appropriate amount of oil (final concentrations of 25, 50, and 100 μl/l PAE) was added onto the inner surface of each Petri dish lid, and the dishes were quickly covered. Two perpendicular diameters (in cm) of the colony zone were measured with calipers. Each treatment contained three replicates, and the experiment was repeated three times.

### Treatment and Storage of Sweet Potatoes

Sweet potatoes at the postharvest stage were surfaced-disinfected with 75% ethanol and then artificially wounded once to a depth of 10 or 5 mm in diameter. A suspension of *C. fimbriata* at 10^6^ spore/ml (20 μl) was inoculated into each wound. After drying, the inoculated sweet potatoes were randomly distributed into four groups (a control and three PAE vapor treatments). The control groups did not receive PAE.

During the PAE vapor treatments, 25, 50, and 100 μl PAE were placed in 1 l polystyrene containers with snap-on lids to allow for natural evaporation (Tian et al., 2011). The vapor concentration was the ratio of the volumes of the PAE and the containers (μl/l). Hence, the PAE vapor concentrations used in the experiment were 25, 50, and 100 μl/l air.

### *In Vivo* Assay

The treatment and storage methods were the same as those detailed in the Section ‘Treatment and Storage of Sweet Potatoes.’ For the treated groups, each sweet potato was placed into a 1 l container. Based on our preliminary experiments, 25, 50, or 100 μl/l PAE were placed in a small beaker and then in the sealed container. The PAE was spontaneously volatilized inside the residual airspace of the containers at 28°C for 21 days. Lesion diameter was expressed as the mean width and length of the areas of decay (Shao et al., 2013). Each treatment involved three replications, and the entire experiment was conducted in triplicate.

### Effects of *C. fimbriata* on Defense-Related Enzyme Activity in Sweet Potatoes

To evaluate the elicitation of active defense responses via PAE vapor treatments, tissue samples surrounding each wound in the fruit were collected at 0, 3, 6, 9, 12, 15, 18, and 21 days in each group.

All enzyme extraction procedures were conducted at 4°C. The tissues around the wounds of the sweet potatoes were collected using a sterilized knife and then homogenized to determine their defense-related enzyme activity. The extracts were then homogenized and centrifuged at 12,000 × *g* for 30 min at 4°C. The supernatant was used for the enzyme assay.

Superoxide dismutase was extracted using a modification of the method used by Liu et al. (2005), Vattem et al. (2005), and Li et al. (2017) and determined via nitro-blue tetrazolium (NBT) reaction. Five g of fresh sample (homogenized sweet potatoes) were ground with 5 ml of 0.1 M sodium phosphate buffer (pH 7.8). The absorbance at 560 nm was recorded.

Catalase was extracted using a slight modification of the protocol used by Cao et al. (2008). Homogenized sweet potatoes (5 g) were ground with 5 ml of 0.1 M sodium phosphate buffer (pH 7.5). CAT activity was determined by adding 0.1 ml of the enzyme preparation to 2.9 ml of 20 mM hydrogen peroxide (H_2_O_2_), which acted as the substrate. One unit was defined as the change in 0.01 absorbance units per minute at 240 nm, as determined with a UV-visible spectrophotometer.

For the POD extraction, fresh sample (5 g of homogenized sweet potatoes) was ground with 5 ml of 0.1 M sodium acetate buffer (pH 5.5) containing 4% polyvinylpolypyrrolidone (PVPP) (m/v), 1 mM polyethylene glycol (PEG) (m/v), and 1% Trition X-100 (v/v). POD activity was determined via the method of Shao et al. (2013). Enzyme activity was defined as the increase in absorbance, and one unit was defined as the change in absorbance units per minute at 420 nm, as determined with a UV-visible spectrophotometer.

Five gram of homogenized sweet potatoes were ground with 5 ml of 0.1 M potassium phosphate buffer (pH 7.5) for APX extraction (Cao et al., 2008; Li et al., 2016). APX activity was determined by adding 0.1 ml of the enzyme preparation to 2.6 ml of potassium phosphate buffer containing 0.1 mM EDTA and 0.5 mM AsA, as well as adding 0.3 ml of H_2_O_2_, which acted as the substrate. Enzyme activity was defined as the decrease in absorbance, and one unit was defined as the change in 0.01 absorbance units per minute at 290 nm as determined with a UV-visible spectrophotometer.

Polyphenol oxidase extraction and activity determination were carried out according to the method of Shao et al. (2013), with slight modifications. Briefly, 5 g of fresh sample were ground with 5 ml of 0.1 M sodium acetate buffer (pH 5.5) containing 4% PVPP (m/v), 1 mM PEG (m/v), and 1% Trition X-100 (v/v). Enzyme activity was defined as the increase in absorbance, and one unit was defined as the change in 0.1 absorbance units per minute at 420 nm, as measured with a UV-visible spectrophotometer.

Phenylalanine ammonia-lyase was extracted with 0.1 M brax buffer at a pH of 8.8, which contained 40 g/l PVPP (m/v), 2 mM EDTA (m/v), and 5 mM β-mercaptoethanol (v/v). PAL activity was determined according to the method of Assis et al. (2001) and Zeng et al. (2006). One unit was defined as the change in 0.01 absorbance units per hour at 290 nm, as measured with a UV-visible spectrophotometer.

### Determination of Fruit Quality Parameters

Non-inoculated sweet potatoes were randomly distributed into a control and three PAE vapor treatment groups. The method of PAE vapor treatment was described in the Section ‘Treatment and Storage of Sweet Potatoes.’ After treatment, these fruits were released from the PAE vapor and stored at 28°C for 21 days to investigate changes in quality parameters.

At harvest, the fruits were evaluated by taking the following measurements: weight loss, anthocyanin content, and ascorbic acid content. Tissues around the wound of the sweet potatoes were collected using a sterilized knife and then homogenized to determine their anthocyanin content and ascorbic acid content.

Weight loss was expressed as a percentage of total weight. On each day of storage, sweet potatoes from each treatment were weighed, and then, the weight loss percentage was calculated with respect to the initial weight of the sweet potatoes. The results were obtained from three replicates.

Anthocyanin content was measured as described by Mirdehghan and Rahimi (2016). Five gram of homogenized sweet potatoes were centrifuged at 10,000 rpm. Then, hydrochloric acid-potassium chloride (pH = 1) and acetate (pH = 4.5) buffers were used to dilute the supernatants. The absorbance was measured with a UV-Vis spectrophotometer at 520 and 700 nm in two buffers at pH 1 and 4.5, respectively. All concentrations were measured in three replicates, and each experiment was performed three times.

Ascorbic acid content (vitamin C) was measured via titrimetric methods (Kim and Yook, 2009). The method of measuring ascorbic acid utilized 2,6-dichlorophenol indophenol dye. The reduction of this dye by ascorbic acid is specific. Five g of homogenized sweet potatoes were mixed with 100 ml of a mixture of metaphosphoric and acetic acids (30 g of metaphosphoric acid and 80 ml of acetic acid were diluted to 1 l with distilled water). The sample acid mixture (10 ml) was titrated with indophenol (250 mg of sodium carbonate and 250 mg of indophenol were massed up to 1 l of distilled water). Three replicates were conducted for each parameter, and the entire experiment was performed three times.

### Statistical Analysis

The data were analyzed via a one-way analysis of variance (ANOVA), followed by Duncan’s multiple-range tests at *p* < 0.05 (SPSS Statistics 17.0 Inc.). In the statistical analysis of the randomized complete block design, each treatment involved three replications, and the entire experiment was conducted in triplicate.

## Results

### Evaluation of *in Vitro* Antifungal Activity

**FIGURE 1:**
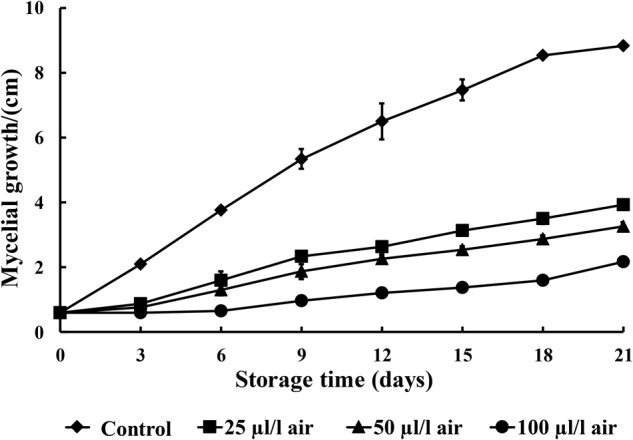
The effects of various concentrations of PAE vapor on the mycelial growth of *C. fimbriata*. Data were the means of three replicates ± SD.

PAE at 25, 50, and 100 μl/l can effectively inhibit the mycelial growth of *C. fimbriata* in PDA medium over 21 days of incubation. In our study, PAE showed a notable antifungal effect on *C. fimbriata* (**Figure 1**). The inhibitory efficacy was enhanced as the PAE concentration increased. The mycelial growth of *C. fimbriata* was moderately inhibited by PAE at a low concentration (25 μl/l). In contrast, 100 μl/l of PAE induced the 100% inhibition of the mycelial growth of *C. fimbriata* for up to 3 days of culture, and the differences between the various PAE concentrations were statistically significant (*p* < 0.05).

### Evaluation of *in Vivo* Antifungal Activity

**FIGURE 2:**
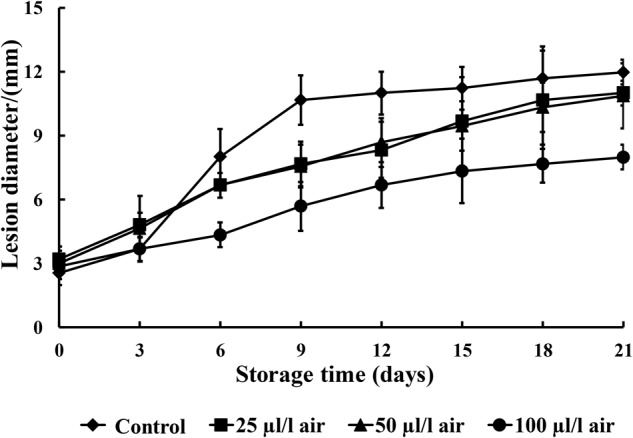
The effects of various concentrations of PAE vapor on the lesion diameter of *C. fimbriata*. Vertical bars represent the standard error of the mean.

**Figure 2** illustrates that all concentrations of PAE reduced the severity of black rot to some extent as compared to the control during the entire storage period. The 100 μl/l PAE concentration group showed significant reductions in lesion diameter from the 6th day onward after inoculation (*p* < 0.05).

### Effect of PAE Vapor Treatment on Defense-Related Enzyme Activities

**FIGURE 3:**
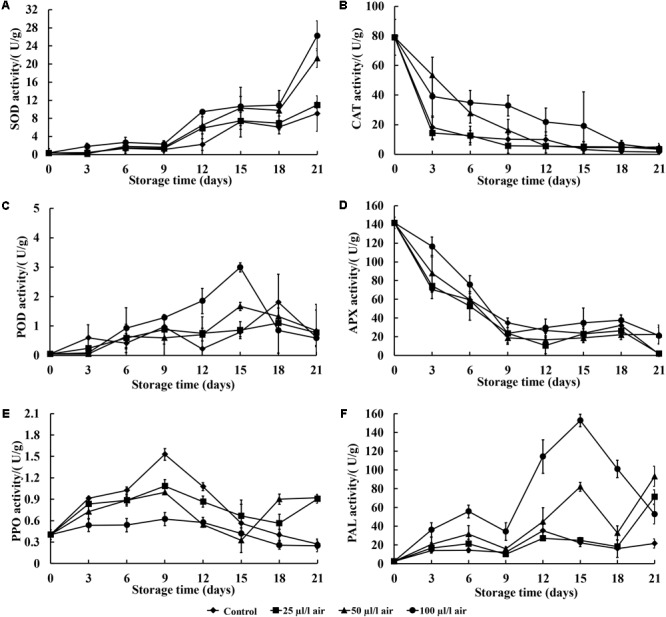
Effect of PAE vapor treatment on SOD **(A)**, CAT **(C)**, APX **(B)**, POD **(D)**, PPO **(E)**, and PAL **(F)** activity levels in sweet potatoes during storage. Values are means ± SD. Vertical bars represent standard errors of the means.

In general, the SOD activity levels of sweet potatoes treated with PAE and the control showed increasing trends, except on the 9th and 18th days after inoculation. There was also a noticeable increase on the 21st day of postharvest storage (**Figure 3A**). The activity levels of SOD in sweet potatoes treated with 100 μl/l PAE were significant higher than those of the control on the 12th and 21st days after inoculation (*p* < 0.05).

**Figure 3B** shows the effect of PAE at different concentrations on CAT activity levels in sweet potatoes inoculated with *C. fimbriata.* In general, the CAT activity levels of all samples decreased sharply during storage, with the PAE-treated sweet potatoes having higher levels of activity as compared to the control.

As demonstrated in **Figure 3C**, the patterns of change in POD activity in the control group and two of the treatment groups (PAE with 25 and 50 μl/l) were similar during storage. Overall, the POD activity levels of all groups generally increased over the entire period, and a notable decrease in POD activity levels in the PAE-treated sweet potatoes was observed at the 21st day post-inoculation. In addition, during the entire storage period, all groups that received PAE vapor treatment showed no significant differences as compared to the control, except on the 15th day (*p* < 0.05).

In terms of APX activity, in general, the PAE vapor treatments led to higher APX activity levels than those seen in the control over the entire incubation period (**Figure 3D**). APX activity levels in all four groups showed a noticeable decrease from the 3rd to the 9th day and then remained steady after this timepoint. Furthermore, the group fumigated with PAE at 100 μl/l showed significantly higher APX activity as compared with the control, except on the 12th, 15th, and 18th days (*p* < 0.05).

**Figure 3E** shows the fluctuations in PPO activity levels, which increased at the 9th day and then decreased in a relatively unstable way during the remaining days. The PPO activity levels of all groups generally went up, and PPO activity in the sweet potatoes given PAE vapor treatments undulated steadily during post-inoculation storage. Meanwhile, in the control group, PPO activity underwent a sharp fluctuation. The PPO activity level was remarkable lower in PAE-treated fruits than in non-treated sweet potatoes, and no significant differences occurred after the 15th day of storage (*p* < 0.05).

Regarding the PAL activity levels of all four groups, **Figure 3F** shows a generally increasing trend with slight fluctuations before the 15th day. In the PAE-treated groups, except for the 25 μl/l PAE group, PAL exhibited significantly higher activity levels than in the control group at 3 days postharvest (*p* < 0.05).

### Effects of PAE Vapor on Fruit Quality Parameters

**FIGURE 4:**
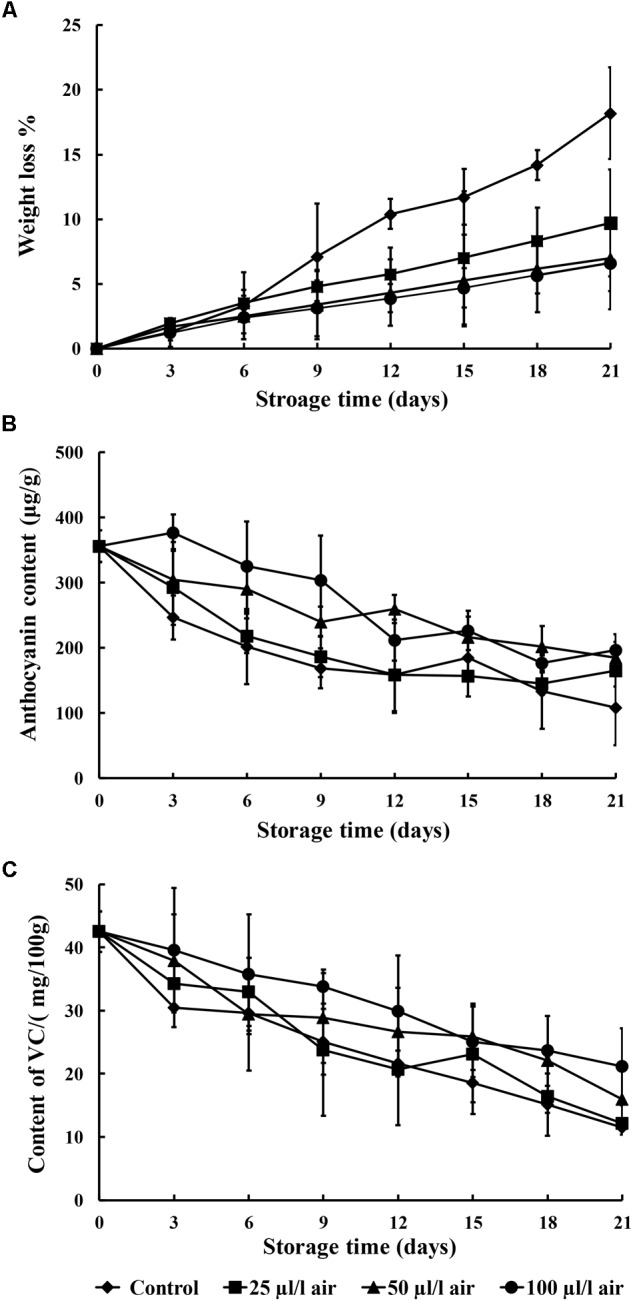
Effect of PAE vapor treatment on weight loss **(A)**, anthocyanin content **(B)**, and vitamin C content **(C)** in sweet potatoes during storage. Values are means ± SD. Vertical bars represent standard errors of the means.

It is apparent from **Figure 4A** that the weight loss in the sweet potatoes increased markedly as the storage period advanced. Among the various PAE concentrations, sweet potatoes fumigated with 100 μl/l PAE exhibited substantially less weight loss during the 21 days of storage as compared to the other treatments. In addition, after the 12th day of storage, the PAE-treated groups showed a significant decrease in weight loss rates as compared to the control (*p* < 0.05).

In our work, the anthocyanin content of the sweet potatoes was shown to decline during the entire storage period. In the control group, it decreased relatively quickly, whereas in the three PAE-treated groups, it decreased gradually over the entire storage period (**Figure 4B**). In addition, the anthocyanin content was higher in sweet potatoes treated with all concentrations of PAE than in the non-treated group.

As shown in **Figure 4C**, the vitamin C content of sweet potatoes fumigated with 25, 50, and 100 μl/l PAE and the control underwent slight variation. The values for the control were significantly reduced as compared to all the treatments, especially samples fumigated with 100 μl/l PAE, at the 21st day of storage. During the entire storage period, sweet potatoes fumigated with 25, 50, and 100 μl/l PAE had higher vitamin C content than the control (*p* < 0.05).

## Discussion

The postharvest decay of fruits and vegetables causes considerable losses during storage, and 20–25% of harvested fruits and vegetables are decayed by pathogens during the postharvest period (Sharma et al., 2009). The application of synthetic fungicides for the control of pathogenic fungi is a standard commercial practice worldwide; however, because of the increasing awareness of chemical compounds that are potentially harmful to human health and the environment, interest in natural methods of maintaining postharvest quality and controlling diseases in plants is increasing (Sharma et al., 2009). Essential oils, as biologically active agents, represent rich potential sources of alternative and environmentally acceptable compounds for disease management (Servili et al., 2017). PAE, a major constituent of essential oil, is “generally regarded as safe” (GRAS) by the United States Food and Drug Administration (Hobbs et al., 2016) and could be developed into a natural preservative for controlling the infection of sweet potatoes by spoilage fungi.

In our study, the antifungal activities of PAE vapor on fungal mycelial growth were assessed. PAE vapor treatment can remarkably reduce the mycelial growth of *C. fimbriata in vitro*. Thus, these *in vitro* results confirm the effectiveness of PAE as an antifungal agent against *C. fimbriata* and reveal that *C. fimbriata* is sensitive to PAE vapor in a dose-dependent manner. To further provide proof-of-concept that PAE vapor is active against black rot caused by the pathogenic fungus *C. fimbriata*, we conducted *in vivo* investigations to assess its efficacy as a natural preservative for the control of decay in sweet potatoes. In this *in vivo* experiment, the PAE vapor treatments also alleviated black rot in artificially infected sweet potatoes.

The activation of defense-related enzymes in fruit is considered to be important in conferring resistance against postharvest diseases (Tian et al., 2006; Wang et al., 2014). One of the most prominent plant defense responses is an oxidative burst, or an accumulation of reactive oxygen species (ROS) (Foyer and Noctor, 2011; Zheng et al., 2017). The generation of ROS serves as a signal that activates additional plant defense reactions (Perumal et al., 2017). Antioxidant and ROS-scavenging systems can effectively help to protect plants from free radicles and stabilize these free radicals (Cao et al., 2008). Generally, ROS are controlled by an array of antioxidant enzymes, such as SOD, CAT, and APX (Deng et al., 2015). They are considered the key enzymes in host defense reactions against pathogenic infections (Zhang et al., 2011; Ma et al., 2013). As the first line of defense against the damages caused by oxygen radicals, SOD is a metalloprotein that catalyzes the dismutation of O_2_^-^ into molecular oxygen and H_2_O_2_, while CAT converts H_2_O_2_ into oxygen and water (Sellamuthu et al., 2013). Therefore, the increased antioxidant enzyme activity levels (SOD, CAT, and APX) in the PAE-vapor-treated sweet potatoes can protect the cell membrane structure and function of the sweet potato tissue by inhibiting the accumulation of reactive oxygen species, resulting in less oxidative stress and damage to the sweet potatoes and thereby contributing to the fruit tissue’s resistance against *C. fimbriata*.

In addition to SOD, CAT, and APX activity levels, POD, PAL, and PPO activity levels also play an important role in inducing resistance in fruits (Tian et al., 2006; Yang et al., 2017). POD activity produces the oxidative power needed for the cross-linking of proteins and phenylpropanoid radicals, resulting in the reinforcement of cell walls against fungal penetration (Yao and Tian, 2005). Previous researchers have also suggested that POD is related to enhanced disease resistance in plants (Mohammadi and Kazemi, 2002; Qin et al., 2003; Zhang et al., 2011). PAL is a key enzyme involved in the first step of propane metabolism, which is related to the plant defense system (Dixon et al., 2002; Yao and Tian, 2005; Liu et al., 2016). Also, PAL directly participates in the synthesis of active metabolites associated with plant protection and the local resistance process, including phenols and lignin (Cao et al., 2008). In our study, we confirmed significantly enhanced PAL activity in response to PAE vapor treatment. PPO is a copper enzyme that can catalyze several reactions leading to the formation of quinones. Quinone synthesis is one of the first responses to fungal attack or wounding (Cindi et al., 2016). In this study, PAE vapor treatment was found to alter PPO activity during incubation, which may result in enhanced pathogen resistance in sweet potatoes. Thus, it seems that these effects could collectively contribute to the development of disease resistance against *C. fimbriata*.

During the postharvest storage of sweet potatoes, changes related to quality, such as the weight loss rate, anthocyanin content, and vitamin C content, were generally observed. The weight loss rate is an important fruit quality parameter during storage (Castillo et al., 2014; Tao et al., 2014). Weight loss is associated with the absence of the protective epidermal layer and waxes, resulting in the deterioration of quality (Toivonen and Brummell, 2008). Our results indicate that PAE can maintain high-quality postharvest sweet potatoes. Anthocyanins, as water-soluble pigments, occur in fruits and vegetables and play important roles in protecting plants against various biotic and abiotic stresses (Mirdehghan and Rahimi, 2016). Our study suggests that PAE vapor can enhance anthocyanin accumulation. Vitamin C is the water-soluble vitamin that is most sensitive to irradiation, and it is also highly sensitive to various modes of degradation (Kilcast, 1994). PAE vapor treatment led to fruits with higher ascorbic acid content at harvest and during postharvest storage. Thus, postharvest PAE vapor treatment can improve the quality and storability of harvested sweet potatoes.

## Conclusion

The aim of this study was to determine the effectiveness of PAE in controlling postharvest decay in sweet potatoes. PAE vapor significantly reduced *C. fimbriata*, the main pathogen affecting postharvest sweet potatoes, both *in vitro* and *in vivo*. PAE vapor inhibited artificially inoculated black rot caused by *C. fimbriata* and helped maintain the weight loss rate, anthocyanin content, and vitamin C content in postharvest sweet potatoes, which suggests that PAE could be a potential method of enhancing anthocyanin and vitamin C accumulation and maintaining high-quality postharvest sweet potatoes. In addition, PAE vapor treatment may enhance the resistance of postharvest sweet potatoes to *C. fimbriata* through several defense-related enzymes (SOD, CAT, APX, POD, PPO, and PAL). Taken together, the ability of PAE to reduce decay in postharvest sweet potatoes may be associated with the elicitation of the host defense response. These results suggest that the mode of action of PAE appears to occur both via direct interaction with the fungus itself and via defensive responses in the fruit tissue. Hence, the postharvest application of PAE is a promising strategy for the control of postharvest diseases in sweet potatoes. In addition, further experiments are required to investigate the influence of PAE on global transcriptional changes in sweet potatoes using RNA-Seq technology.

## Author Contributions

JT and YL designed the experiments. MZ and ML performed the experiments. CP and SP analyzed the data. MZ and ML drafted the manuscript. All authors read and approved the final manuscript.

## Conflict of Interest Statement

The authors declare that the research was conducted in the absence of any commercial or financial relationships that could be construed as a potential conflict of interest.
